# A Missense Variant in *KCNJ10* in Belgian Shepherd Dogs Affected by Spongy Degeneration with Cerebellar Ataxia (SDCA1)

**DOI:** 10.1534/g3.116.038455

**Published:** 2016-12-21

**Authors:** Nico Mauri, Miriam Kleiter, Michael Leschnik, Sandra Högler, Elisabeth Dietschi, Michaela Wiedmer, Joëlle Dietrich, Diana Henke, Frank Steffen, Simone Schuller, Corinne Gurtner, Nadine Stokar-Regenscheit, Donal O’Toole, Thomas Bilzer, Christiane Herden, Anna Oevermann, Vidhya Jagannathan, Tosso Leeb

**Affiliations:** * Institute of Genetics, and Vetsuisse Faculty, University of Bern, 3001, Switzerland; §§§ Division of Neurological Sciences, Department of Clinical Research and Veterinary Public Health,; ‡‡ Division of Small Animal Internal Medicine, Department of Clinical Veterinary Medicine,; ** Division of Clinical Neurology, Department of Clinical Veterinary Medicine, Vetsuisse Faculty, University of Bern, 3001, Switzerland; †† Section of Neurology, Department of Small Animals, Vetsuisse Faculty, University of Zurich, 8057, Switzerland; §§ Institute of Animal Pathology, Department of Infectious Diseases and Pathobiology, Vetsuisse Faculty, University of Bern, 3001, Switzerland; †,‡ University Clinic for Small Animals, Department for Companion Animals and Horses,; § Institute of Pathology and Forensic Medicine, Department of Pathobiology, University of Veterinary Medicine Vienna, 1210, Austria; *** Wyoming State Veterinary Laboratory, University of Wyoming, Laramie, Wyoming 82070; ††† Institute of Neuropathology, University Hospital Düsseldorf, 40225, Germany; ‡‡‡ Institute of Veterinary Pathology, Justus-Liebig-University, 35392 Gießen, Germany

**Keywords:** *Canis familiaris*, Kir4.1, potassium channel, EAST syndrome, SeSAME syndrome, Malinois, neurology, brain, central nervous system, animal model

## Abstract

Spongy degeneration with cerebellar ataxia (SDCA) is a severe neurodegenerative disease with monogenic autosomal recessive inheritance in Malinois dogs, one of the four varieties of the Belgian Shepherd breed. We performed a genetic investigation in six families and seven isolated cases of Malinois dogs with signs of cerebellar dysfunction. Linkage analysis revealed an unexpected genetic heterogeneity within the studied cases. The affected dogs from four families and one isolated case shared a ∼1.4 Mb common homozygous haplotype segment on chromosome 38. Whole genome sequence analysis of three affected and 140 control dogs revealed a missense variant in the *KCNJ10* gene encoding a potassium channel (c.986T>C; p.Leu329Pro). Pathogenic variants in *KCNJ10* were reported previously in humans, mice, and dogs with neurological phenotypes. Therefore, we consider *KCNJ10*:c.986T>C the most likely candidate causative variant for one subtype of SDCA in Malinois dogs, which we propose to term spongy degeneration with cerebellar ataxia 1 (SDCA1). However, our study also comprised samples from 12 Malinois dogs with cerebellar dysfunction which were not homozygous for this variant, suggesting a different genetic basis in these dogs. A retrospective detailed clinical and histopathological analysis revealed subtle neuropathological differences with respect to SDCA1-affected dogs. Thus, our study highlights the genetic and phenotypic complexity underlying cerebellar dysfunction in Malinois dogs and provides the basis for a genetic test to eradicate one specific neurodegenerative disease from the breeding population. These dogs represent an animal model for the human EAST syndrome.

EAST syndrome in humans is characterized by epilepsy, ataxia, sensorineural deafness, and renal salt wasting tubulopathy (Bockenhauer *et al.* 2009; OMIM#612780). It was also termed SeSAME syndrome for seizures, sensorineural deafness, ataxia, mental retardation, and electrolyte imbalance (Scholl *et al.* 2009). EAST syndrome is a rare disorder with an autosomal recessive mode of inheritance. Until now, it has been reported in 26 human patients with a total of 14 different pathogenic variants in the *KCNJ10* gene (Sala-Rabanal *et al.* 2010; Williams *et al.* 2010; Abdelhadi *et al.* 2016).

*KCNJ10* encodes an inward-rectifying potassium channel (K_ir_), also known as Kir4.1, which is expressed in the central nervous system (CNS), eye, inner ear, and kidney. In the kidneys, this channel is located in the distal nephron’s basolateral tubular epithelia. The KCNJ10 channels in the intermediate cells of the stria vascularis of the inner ear contribute to the endocochlear potential (Bockenhauer *et al.* 2009; Abdelhadi *et al.* 2016; Palygin *et al.* 2016). In the eye, KCNJ10 is expressed in Müller glia cells (Sala-Rabanal *et al.* 2010; Arai *et al.* 2015). Brain expression predominates in the cerebral and cerebellar cortex as well as in the caudate nucleus and putamen. KCNJ10 channels play a major role in modulating neuronal cells’ resting membrane potential through a process named potassium spatial buffering. Potassium spatial buffering, ensured by glial cells, mainly astrocytes, is a mechanism for the regulation of extracellular potassium (Djukic *et al.* 2007; Bockenhauer *et al.* 2009; Scholl *et al.* 2009; Sala-Rabanal *et al.* 2010).

The clinical hallmarks of EAST syndrome are nonprogressive neurological and renal symptoms. They include infantile-onset seizures, ataxia, tubulopathy, and sensorineural deafness (Bockenhauer *et al.* 2009; Cross *et al.* 2013; Abdelhadi *et al.* 2016). In the kidneys, KCNJ10 defects result in renal salt wasting and hypokalemic metabolic alkalosis (Bockenhauer *et al.* 2009; Abdelhadi *et al.* 2016). Additionally, the failure to generate a physiologic endocochlear potential can result in sensorineural deafness. Ocular abnormalities in EAST syndrome are only detectable with electroretinogram and are characterized by reduced amplitudes of the photopic negative response of light-adapted retinae (Thompson *et al.* 2011; Arai *et al.* 2015). *Kcjn10*-deficient knockout mice show defects in oligodendrocyte development and *in vivo* myelination (Neusch *et al.* 2001; Djukic *et al.* 2007).

A *KCNJ10* variant has also been reported in dogs. In several terrier breeds, the XM_545752.3:c.627C>G variant, predicted to result in p.Ile209Met, leads to hereditary ataxia, a disease characterized by lesions in the CNS, mainly in the spinal cord, but not in the cerebellum (Wessmann *et al.* 2004; Rohdin *et al.* 2010, 2015; Gilliam *et al.* 2014).

In the Belgian Shepherd breed, spongy degeneration with cerebellar ataxia (SDCA) was first described in 13 purebred Malinois puppies from five different litters. Clinical signs and histological findings were primarily localized to the cerebellum. The pedigree data suggested an autosomal recessive mode of inheritance (Kleiter *et al.* 2011). An earlier report of two cross-bred Malinois dogs may have described a similar disease (Cachin and Vandevelde 1991). However, to date, no causal variant for SDCA was described in Malinois dogs or any other of the varieties of the Belgian Shepherd breed.

The aim of this study was to identify the presumed causative genetic defect of SDCA in Malinois dogs using a positional cloning approach in combination with whole genome resequencing. The genetic analysis revealed an unexpected genetic heterogeneity and our findings strongly suggest that in the Belgian Shepherd breed more than one type of cerebellar dysfunction is present.

## Materials and Methods

### Ethics statement

All animal experiments were performed according to the local regulations. All dogs in this study were examined with the consent of their owners. The collection of blood samples was approved by the “Cantonal Committee for Animal Experiments” (Canton of Bern; permit 23/10).

### Breed nomenclature

The Federation Cynologique Internationale (FCI) describes the Malinois, together with the Groenendael, the Laekenois, and the Tervueren, as a variety of the Belgian Shepherd dog breed. The American Kennel Club, however, officially recognizes the Belgian Malinois, the Belgian Sheepdog (FCI: Groenendael), the Belgian Laekenois, and the Belgian Tervuren (FCI: Tervueren) as four distinct breeds. In this paper, all references to the breed nomenclature correspond to the FCI standards.

### Clinical examinations

**Figure 1:**
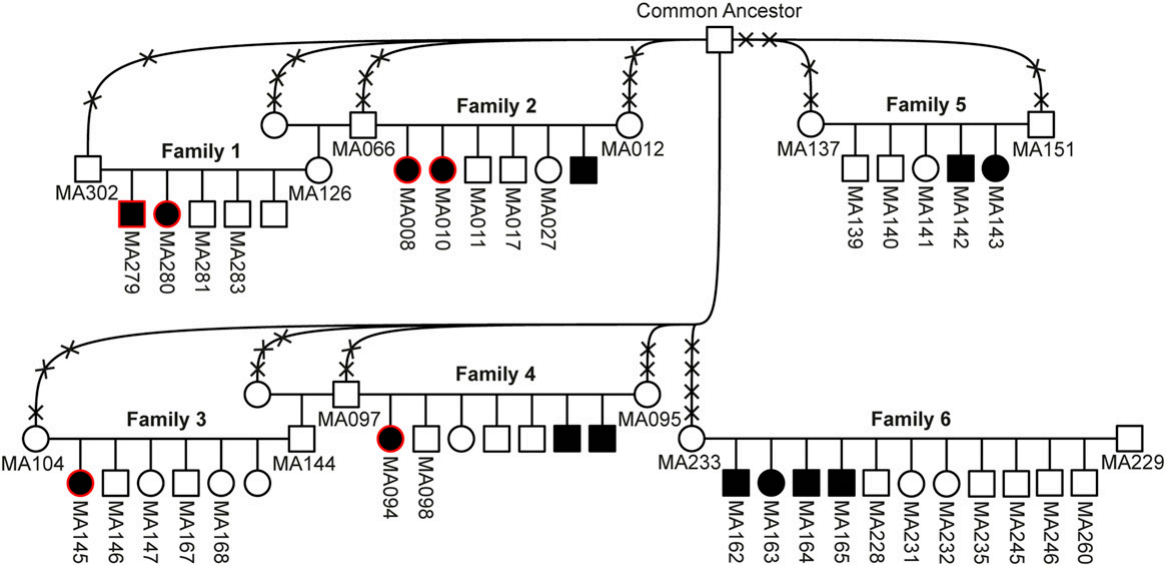
Pedigree of Malinois dogs used for genetic mapping of the disease locus. Filled symbols represent animals with cerebellar disorder. Numbers indicate dogs from which samples were available and which were genotyped on the SNP chip for linkage analysis. Six affected dogs indicated by red contours were selected for homozygosity mapping. A common ancestor in both maternal and paternal lineages could be identified in five of the six families. This dog had >1500 descendants in just three generations. Crosses intersecting the connection lines to the common ancestor represent the numbers of generations (*e.g.*, MA302 is a great-grandson of the common ancestor). Family 4 was previously described (Kleiter *et al.* 2011).

In this study, we performed neurological examination including video analysis of 12 Malinois puppies with clinical signs of cerebellar disease. These dogs belonged to six related families (Figure 1). Family 4 in the pedigree was one of the five families previously reported with SDCA (Kleiter *et al.* 2011).

### Neurohistopathology examinations

Necropsy was performed in nine out of the 12 above described Malinois puppies with neurological symptoms (MA008, MA094, MA142, MA143, MA145, MA162, MA164, MA165, and MA280, Figure 1). We collected brain samples from all nine and spinal cord samples from seven puppies (MA142, MA143, MA145, MA162, MA164, MA165, and MA280). All tissues were fixed in 4% buffered formaldehyde solution, embedded in paraffin, and sectioned at 2–5 µm. Sections were stained with hematoxylin and eosin and examined by light microscopy.

### Animal selection for the genetic analysis

For the genetic analysis, we initially applied liberal phenotypic inclusion criteria and considered all available Malinois dogs with neurological abnormalities. Specifically, we used six Malinois families. Samples were available from the 12 Malinois puppies with cerebellar disorder, 20 nonaffected full siblings, and 12 nonaffected parents (Figure 1). We additionally investigated seven Malinois puppies with reported cerebellar signs, for which no relatives were available.

In addition to the individuals belonging to the six families and the seven isolated cases, we genotyped 187 other Malinois, 25 Groenendael, two Laekenois, and 34 Tervueren dogs whose blood samples were donated to the biobank of the Institute of Genetics at the University of Bern (Table 2). Furthermore, we analyzed 486 samples from 89 genetically diverse dog breeds (Supplemental Material, Table S1).

### DNA extraction and genotyping

Genomic DNA was isolated from EDTA blood samples with the Maxwell RSC Whole Blood DNA Kit, which were used with the Maxwell RSC Instrument (Promega). Genotyping was done on Illumina canine_HD chips containing 173,662 genome-wide single nucleotide polymorphisms (SNPs) by GeneSeek/Neogen. Genotypes were stored in a BC/Gene database version 3.5 (BC/Platforms).

### Linkage and homozygosity mapping

For linkage analysis, Illumina canine_HD SNP chip genotypes from 44 dogs in six families were used (Figure 1). We analyzed the dataset for parametric linkage under a fully penetrant, recessive model of inheritance with the Merlin software (Abecasis *et al.* 2002).

PLINK v1.07 (Purcell *et al.* 2007) was used as described (Wiedmer *et al.* 2016) to search for extended intervals of homozygosity with shared alleles across the affected animals.

### Reference sequences

The dog CanFam 3.1 genome assembly was used for all analyses. All references to the canine *KCNJ10* gene correspond to the accessions XM_545752.5 (mRNA) and XP_545752.3 (protein). XP_545752.3 has the same length as the human protein (NP_002232.2; 379 amino acids) and 373 out of 379 amino acids (98%) are identical between dog and human.

### Whole genome resequencing

PCR-free fragment libraries were prepared from three affected Malinois dogs (MA008, MA094, and MA152) with 300 bp (MA008, MA094) and 400 bp (MA152) insert sizes. The libraries were sequenced to roughly 16×–22× coverage on an Illumina HiSeq2000 (MA008, MA094) or on an Illumina HiSeq3000 (MA152) instrument using 2 × 100 bp paired-end reads (MA008, MA094) or 170 bp + 130 bp paired-end reads (MA152), respectively.

The reads were mapped to the dog reference genome assembly CanFam3.1 and aligned using Burrows-Wheeler Aligner version 0.7.5a with default settings (Li and Durbin 2009). The generated SAM file was converted to a BAM file and the reads were sorted using samtools (Li 2011). Picard tools (http://sourceforge.net/projects/picard/) was used to mark PCR duplicates. To perform local realignments and to produce a cleaned BAM file, we used the Genome Analysis Tool Kit (GATK version 2.4.9, 50; McKenna *et al.* 2010). GATK was also used for base quality recalibration with canine dbSNP data as training set.

Putative single nucleotide and small indel variants were identified in each sample individually using GATK HaplotypeCaller in gVCF mode, and subsequently genotyped per-chromosome and genotyped across all samples simultaneously (Van der Auwera *et al.* 2013). Filtering was performed using the variant filtration module of GATK. To predict the functional effects of the called variants, SnpEff software together with the ENSEMBL (version 72) annotation CanFam 3.1 was used (Cingolani *et al.* 2012). For variant filtering we used 140 control genomes, which were either publicly available (Bai *et al.* 2015) or produced during other projects of our group.

### PCR and Sanger sequencing

Sanger sequencing was used to confirm the variant identified from whole genome sequencing. For these experiments we amplified PCR products from genomic DNA using AmpliTaqGold360Mastermix (Life Technologies). The PCR primers used for the genotyping of the *KCNJ10:*c.986T>C variant were AGCTGGTGCTGATCCTCAGT (forward primer) and TCCCTTAACGACTCCTCCAA (reverse primer). PCR products were directly sequenced on an ABI 3730 capillary sequencer (Life Technologies) after treatment with exonuclease I and shrimp alkaline phosphatase. Sanger sequence data were analyzed with Sequencher 5.1 (GeneCodes).

### Data availability

File S1 is a video illustrating the clinical phenotype of an affected Malinois dog with the *KCNJ10:*c.986T>C variant at 5, 7, and 8 wk of age (MA008). Table S1 contains *KCNJ10:*c.986T>C genotypes of 486 control dogs from 89 diverse dog breeds. Table S2 lists genome regions ≥1 Mb that showed positive log of odds (LOD) scores in the linkage analysis. Table S3 illustrates the homozygous genome regions with shared alleles among the six analyzed affected Malinois puppies from families 1 to 4 that exhibited phenotypic homogeneity. Table S4 lists the accession numbers of all whole genome sequencing data, which were deposited in the European Nucleotide Archive. Table S5 shows the 23 variants in the critical interval on chromosome 38 that were absent from 140 other dog genomes.

## Results

### Clinical presentation

The 12 Malinois puppies with cerebellar dysfunction had early onset of clinical signs (4.5–8.5 wk of age). During presentation 10 puppies were bright, alert, and responsive. Two puppies were less alert but were responsive. All puppies showed a wide-based ataxic gait, which was more obvious in the hind limbs. Exaggerated gait movements were observed in 50% of the affected puppies. Less consistent clinical signs were stumbling, staggering, intention tremor, bunny hopping, as well as balance loss and falling. Decelerated eye ball coordination during fast head motion was noted in three puppies. Circling episodes or short episodes of muscle spasms together with aggravation of cerebellar symptoms were reported in two puppies after stress or exercise (File S1). The four affected Malinois puppies belonging to family 6 were reported to have seizures, to run into obstacles, and to show rapid progression of clinical signs. Due to the severity of the clinical signs all affected puppies were euthanized by the 17th wk of age.

### Neuropathological findings

Lesions in the cerebellum and brain stem of four Malinois puppies belonging to families 1–4 were similar to those described in Kleiter *et al.* (2011). In these puppies, we observed mild to marked vacuolation of the cerebellar nuclei and granule cell layer, as well as vacuoles and spheroids in the reticular formation. In two of these four puppies, the spinal cord was examined and showed vacuoles and spheroids in the white matter as well.

The affected puppies belonging to family 6 had comparable vacuolation of the cerebellar nuclei and the reticular formation. However, no vacuoles were detected in the cerebellar granule cell layer and no spheroids were seen in the reticular formation. In contrast to the Malinois puppies belonging to families 1–4, all affected puppies of family 6 additionally showed necrotic neurons and marked gliosis in the spinal cord gray matter.

The neuropathological changes noted in family 5 differed from the other families. The two affected puppies in this family showed very few vacuoles in the CNS, but marked gliosis in the cerebellar nuclei, in selected medullary nuclei, and in the spinal cord gray matter.

### Pedigree analysis

Pedigrees were available from six Malinois families and were consistent with a monogenic autosomal recessive mode of inheritance. A common ancestor in both maternal and paternal lineages was identified in five of the six studied families within a maximum of five generations. In the remaining family (family 6), this common ancestor was found on the maternal side, but not on the paternal side (Figure 1).

### Genetic mapping of the causative variant

The mapping of the causative locus was performed by investigating six Malinois families with a total of 12 Malinois puppies with cerebellar dysfunction, 20 nonaffected offspring, and 12 nonaffected parents (Figure 1).

An initial linkage analysis including all animals did not reveal any linked segments. As the histopathological examinations had already suggested subtle phenotypic differences between some of the families, we then performed the linkage analysis separately for each of the six families (Table S2). This revealed that families 1–4 shared two tentatively linked intervals on chromosomes 8 and 38, both reaching a LOD score of 2.449. We assumed that the lack of linkage to chromosome 8 and/or 38 in families 5 and 6 was due to locus heterogeneity and that the affected puppies in these families had possibly genetically distinct diseases.

**Figure 2:**
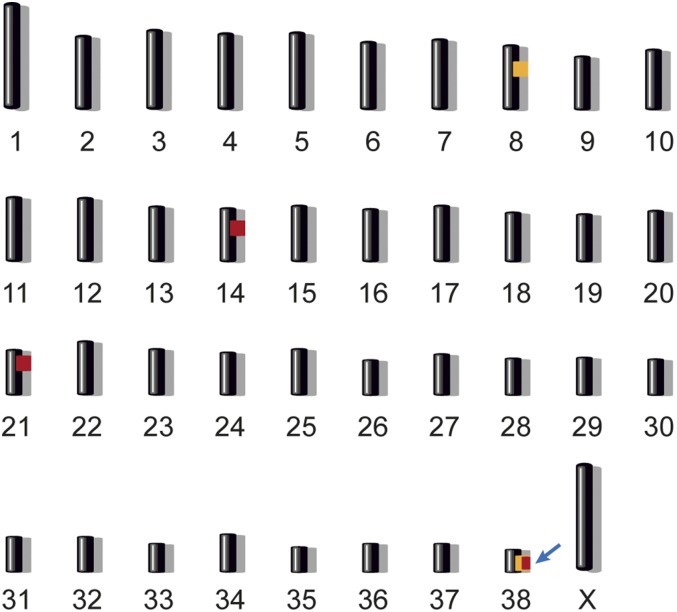
Combined linkage and homozygosity mapping. We performed parametric linkage analysis for a recessive trait in families 1–4 and homozygosity analysis across six selected cases. Two linked genome segments are indicated in orange and three homozygous segments with shared alleles are indicated in red. Only one region on chromosomes 38 showed both linkage and homozygosity and was considered the critical interval (arrow). Specifically, this ∼1.4 Mb region corresponded to Chr38:21,060,597–22,475,242.

Based on the results from the parametric linkage analysis and the pedigree records we hypothesized that the six affected puppies from families 1 to 4 most likely were inbred to one single founder animal. Under this scenario the affected individuals were expected to be identical by descent for the causative genetic variant and flanking chromosomal segments. We used a homozygosity mapping approach to fine-map the region of interest and analyzed the six affected Malinois puppies for extended regions of homozygosity with simultaneous allele sharing. We identified three genome regions with a total of 2.74 Mb that fulfilled our search criteria (Table S3). By intersecting the linked segments from families 1 to 4 and the homozygous intervals from the six cases of these families, only one segment on chromosome 38 remained (Figure 2). Therefore, the combined linkage and homozygosity analysis defined an exact critical interval of 1,414,646 bp at Chr38:21,060,597–22,475,242. Upon inspection of the SNP chip genotypes of isolated Malinois cases with unknown relationships to our families, we identified one additional puppy, which also carried the disease-associated haplotype in homozygous state (MA152). We therefore assumed that this puppy was affected by the same genetic disease as the cases in family 1–4.

### Identification of the causative variant

A total of 30 genes was annotated in the 1.4 Mb critical interval on chromosome 38. To obtain a comprehensive overview of all variants in this region we resequenced the whole genome of three affected Malinois puppies and called single nucleotide as well as indel variants with respect to the reference genome of a presumably nonaffected Boxer (CanFam 3.1). The genotypes of the affected Malinois puppies were further compared with 140 dog genomes from various breeds that had been sequenced during other studies (Table S4). We hypothesized that the causative variant should be completely absent from all dog breeds in the sample set except the Belgian Shepherd breed. After applying this filter, 23 disease-associated variants including two missense variants remained (Table 1 and Table S5).

**Table 1:** Variants detected by whole genome resequencing of three affected Malinois dogs

Filtering Step	Number of Variants
Variants in the whole genome*^a^*	938,586
Variants in the critical 1.4 Mb interval on chromosome 38	3558
Variants in the critical interval that were absent from 140 other dog genomes	23
Nonsynonymous variants in the whole genome*^a^*	6007
Nonsynonymous variants in the 1.4 Mb critical interval on chromosome 38	48
Nonsynonymous variants in the critical interval, absent from 140 other dog genomes	2

a The sequences were compared to the reference genome (CanFam 3.1) from a Boxer. Only variants that were homozygous in all three affected Malinois puppies (MA008, MA094, and MA152) are reported. Nonsynonymous variants were classified based on the ENSEMBL annotation (version 72).

**Figure 3:**
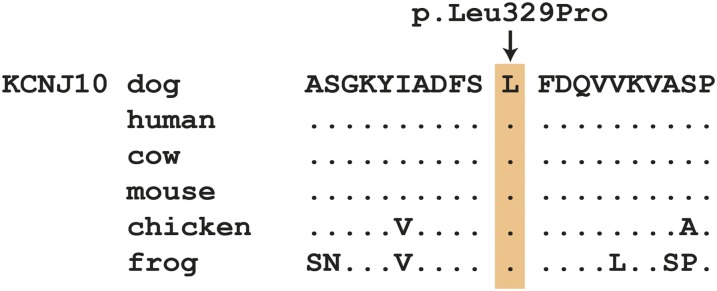
Evolutionary conservation of the leucine residue at position 329 in the KCNJ10 protein. Vertebrates share highly conserved acid sequences in the region of the variant. The sequences were derived from the following database accessions: *C. lupus* XP_545752.3, *H. sapiens* NP_002232.2, *B. taurus* NP_001075070.1, *M. musculus* NP_001034573.1, *G. gallus* XP_003643542.1, *X. tropicalis* NP_001072312.1.

We genotyped the two missense variants in additional dogs and found the variant allele also in healthy control dogs from other breeds. Thus, we considered these variants as unlikely candidates and focused on the remaining 21 variants, which had been predicted to be noncoding by our automated pipeline. When we double-checked these variants with respect to the National Center for Biotechnology Information (NCBI) annotation, we recognized that one, Chr38:22,140,659T>C, is actually a missense variant located within exon 2 of the *KCNJ10* gene, which is not correctly annotated in ENSEMBL. The variant can therefore also be described as *KCNJ10*:c.986T>C and is predicted to lead to a nonconservative amino acid exchange from leucine to proline at codon 329 (p.Leu329Pro). Residue 329 is located in the C-terminal cytoplasmic domain of KCNJ10. The region of the variant is highly conserved across vertebrates (Figure 3).

The presence of this variant in homozygous state was confirmed by Sanger sequencing in the six affected Malinois puppies belonging to families 1–4 and in the isolated case MA152. We additionally genotyped 231 other Malinois, 25 Groenendael, two Laekenois, 34 Tervueren, and 486 dogs of genetically diverse other breeds for this variant. The variant was not found outside the Belgian Shepherd population. The variant was present in heterozygous state or absent in the remaining six cases from families 5 and 6 and the six remaining isolated cases (Table 2 and Table S1).

**Table 2:** Association of the *KCNJ10:*c.986T>C genotypes with cerebellar dysfunction

Genotype *KCNJ10:*c.986T>C	T/T	C/T	C/C
Malinois cases (families 1–4, MA152)	—	—	7
Malinois cases (families 5 and 6, six isolated dogs)	9	3	—
Malinois controls	176	43	—
Groenendael controls	25	—	—
Laekenois controls	2	—	—
Tervueren controls	33	1*^a^*	—
Control dogs from other breeds*^b^*	486	—	—

a This Tervueren dog had Malinois parents.

b These dogs were specifically genotyped for the *KCNJ10:*c.986T>C variant. The genome sequences of 140 independent control dogs were also homozygous T/T at this variant. Therefore, the number of control dogs totals 626.

## Discussion

In this study, we identified a missense variant of *KCNJ10* as a candidate causative genetic defect for SDCA in the Belgian Shepherd breed, more specifically in Malinois dogs. We propose to call this particular phenotype spongy degeneration with cerebellar ataxia, subtype 1 (SDCA1).

We acknowledge the limitations of our linkage analysis, which did not reach a significant LOD score of three in the investigated families. The chosen short read resequencing methodology in combination with an incomplete reference genome was also not 100% sensitive. However, as we identified a nonsynonymous variant in a highly plausible functional candidate gene, we think that our mapping and genome resequencing data combined with the knowledge on *KCNJ10* function in humans, mice, and dogs strongly support the causality of *KCNJ10:*c.986T>C in the Belgian Shepherd breed.

In dogs, a *KCNJ10* variant (XM_545752.3:c.627C>G) has already been reported in several terrier breeds (Gilliam *et al.* 2014; Rohdin *et al.* 2015). This missense variant is predicted to result in p.Ile209Met and leads to hereditary ataxia, also described as spinocerebellar ataxia with myokymia, seizures, or both (SAMS, Gilliam *et al.* 2014). Although there are some similarities in the clinical findings of the terrier’s hereditary ataxia and the SDCA1 in Belgian Shepherd dogs, substantial neuropathological differences in these two distinct diseases are evident. In terriers, the neuropathological lesions are characterized by bilateral symmetrical axonopathy with secondary demyelination, most prominent in the spinal cord. Lesions in the peripheral nervous system and in the brain were also described. However, in contrast to Belgian Shepherd dogs with SDCA1, changes in the cerebellum and/or spongy degeneration in the CNS were not noted in the terrier breeds. Our findings highlight the phenotypic variability caused by different *KCNJ10* variants in the dog, which has also been described in humans (Cross *et al.* 2013; Abdelhadi *et al.* 2016). The two canine variants (p.Ile209Met and p.Leu329Pro) are relatively far apart from each other and also relatively far away from all known human missense variants (Abdelhadi *et al.* 2016). Therefore, a detailed genotype–phenotype correlation with respect to specific amino acid exchanges cannot yet be established. Additional studies are necessary to better understand these rare and complex diseases in dogs and humans.

It remains unclear why seizures were not found in dogs with the *KCNJ10:*c.986T>C variant. However, it is possible that infrequent or subtle (focal) seizures were missed or misinterpreted by owners. Furthermore, future assessment of renal, auditory, and ocular functions in SDCA1-affected Belgian Shepherd dogs could reveal additional similarities to the human EAST syndrome.

Our findings strongly suggest that more than one form of cerebellar dysfunction occurs in the Belgian Shepherd breed. The *KCNJ10:*c.986T>C variant was not present in homozygous state in any of the dogs with cerebellar dysfunction in families 5 and 6, as well as in six isolated Malinois puppies with neurological abnormalities. Moreover, after retrospective revision of histopathology, we identified a subtle phenotype heterogeneity, which further supports our hypothesis of a different genetic basis in closely related litters. However, it also should be kept in mind that neuropathological differences between cases may have been caused by different intervals between onset of clinical signs and postmortem examination.

This study highlights the challenges that dog breeders are confronted with if they do not have access to genetic tests. Prior to our study, there was a strong suspicion that the common ancestor of our six families, a very popular sire, had been a carrier for one recessive neurological defect. Our study demonstrates that the common ancestry of this sire was most likely coincidental and and it remains unclear whether this dog was indeed a carrier for the *KCNJ10* and/or the hypothetical defect(s) underlying the cases in families 5 and/or 6.

In conclusion, we identified the *KCNJ10:*c.986T>C missense variant as most likely causative for SDCA1 in the Belgian Shepherd breed. Cerebellar dysfunction in this breed is heterogeneous and the reported variant explains only a fraction of clinically comparable cases. Further investigations on the other forms of cerebellar dysfunction are needed to clarify their precise phenotype and the underlying genetic variants. The identified affected dogs may serve as a model for the human EAST syndrome. Our findings enable genetic testing in Belgian Shepherd dogs, so that the nonintentional breeding of affected puppies with this specific disease can be avoided in the future.

## Supplementary Material

Supplemental material is available online at www.g3journal.org/lookup/suppl/doi:10.1534/g3.116.038455/-/DC1.
