# Supplementary material for: A Missense Variant in KCNJ10 in Belgian Shepherd Dogs Affected by Spongy Degeneration with Cerebellar Ataxia (SDCA1)
Source: G3 (Bethesda). 2016 Dec 21;7(2):663–9. doi: 10.1534/g3.116.038455 (PMC5295610; doi:10.1534/g3.116.038455)
Supplement: Supplementary file 9 [file 663TableS5.docx]

Table S5. Variants in the critical interval that were absent from 140 other dog genomes. (.xlsx, 14 KB)

<http://www.g3journal.org/lookup/suppl/doi:10.1534/g3.116.038455/-/DC1/TableS5.xlsx>
