# Supplementary material for: A Missense Variant in KCNJ10 in Belgian Shepherd Dogs Affected by Spongy Degeneration with Cerebellar Ataxia (SDCA1)
Source: G3 (Bethesda). 2016 Dec 21;7(2):663–9. doi: 10.1534/g3.116.038455 (PMC5295610; doi:10.1534/g3.116.038455)
Supplement: Supplementary file 11 [file 663FileS1.docx]

File S1. Video illustrating the clinical phenotype of an affected Malinois dog with the *KCNJ10:*c.986T>C variant at five, seven, and eight weeks of age (MA008). (.mp4, 11.65 MB)

<http://www.g3journal.org/lookup/suppl/doi:10.1534/g3.116.038455/-/DC1/FileS1.mp4>
